# Insight into the orientational versatility of steroid substrates—a docking and molecular dynamics study of a steroid receptor and steroid monooxygenase

**DOI:** 10.1007/s00894-017-3278-z

**Published:** 2017-03-01

**Authors:** Anna Panek, Alina Świzdor, Natalia Milecka-Tronina, Jarosław J. Panek

**Affiliations:** 1 Department of Chemistry, Wrocław University of Environmental and Life Sciences, C. K. Norwida 25, 50-375 Wrocław, Poland; 2 Faculty of Chemistry, University of Wrocław, F. Joliot-Curie 14, 50-383 Wrocław, Poland

**Keywords:** Steroids, Progesterone, DHEA, Baeyer–Villiger monooxygenase, Molecular docking, Molecular dynamics

## Abstract

Numerous steroids are essential plant, animal, and human hormones. The medical and industrial applications of these hormones require the identification of new synthetic routes, including biotransformations. The metabolic fate of a steroid can be complicated; it may be transformed into a variety of substituted derivatives. This may be because a steroid molecule can adopt several possible orientations in the binding pocket of a receptor or an enzyme. The present study, based on docking and molecular dynamics, shows that it is indeed possible for a steroid molecule to bind to a receptor binding site in two or more orientations (normal, head-to-tail reversed, upside down). Three steroids were considered: progesterone, dehydroepiandrosterone, and 7-oxo-dehydroepiandrosterone. Two proteins were employed as hosts: the human mineralocorticoid receptor and a bacterial Baeyer–Villiger monooxygenase. When the steroids were in nonstandard orientations, the estimated binding strength was found to be only moderately diminished and the network of hydrogen bonds between the steroid and the host was preserved.

## Introduction

Steroid hormones and their derivatives form a large group of useful pharmaceutical preparations that are employed in the prevention and treatment of diverse diseases in gynecology, endocrinology, rheumatology, oncology, etc. They are used medically and industrially because numerous steroids are essential plant, animal, and human hormones. Compounds such as hydrocortisone, dehydroepiandrosterone (DHEA), and prednisolone are among the best-known steroid drugs and food additives. Brassinosteroids (plant growth factors) are used to boost crop yields [1]. Medical and industrial applications of this class of compounds require the identification of new synthetic routes, preferably using cheap starting materials (e.g., phytosterols). Such routes include biotransformations, which are ecologically friendlier and more stereospecific synthetic pathways than chemical derivatization [2]. However, it is not easy to predict which particular derivatives can be obtained using bacterial or fungal organisms, or even whether a particular species of microorganism can transform an available substrate at all. Moreover, the metabolic fate of steroids is frequently found to be complicated: a given compound can be transformed into a variety of substituted derivatives. For example, the biotransformation of pregnenolone in cultures of the filamentous fungus *Penicillium camemberti* AM83 yields five different derivatives, including progesterone, androstenedione, and dehydroepiandrosterone (DHEA) [3]. The diversity of the metabolic routes of steroids indicates that they are transformed by several groups of enzymes. The conversion of cholesterol by *Mycobacterium* sp. Ac-1815D leads to at least six products; the enzymes involved are 3β-hydroxysteroid and 3(17)-hydroxysteroid dehydrogenases, 3-ketosteroid 1,2-dehydrogenase, and side-chain degradation enzymes [4]. Side-chain degradation involves four groups of inducible enzymes: the fatty acid β-oxidation, ω-oxidase reaction, methyl-crotonyl-CoA carboxylation, and propionyl-CoA carboylase systems [5]. However, in some cases where only one enzyme appears to be involved, an alternative explanation for the diversity of steroid derivatives has been suggested. An extensive study on transformations of acetylaminosteroids [6] revealed that some hydroxylations carried out by the fungus *Curvularia lunata* can be rationalized by assuming that the steroid molecule can adopt several possible orientations in the enzymatic binding pocket. The idea that a steroid ligand can adopt multiple orientations in a binding pocket was investigated in 1967 in a study of the steroid conversion capabilities of *Aspergillus tamarii* [7], where at least four such orientations were proposed. This mechanism can also partially account for the broad range of reactions carried out by one of most popular whole-cell fungal biocatalysts, *Rhizopus oryzae* [8]. A simple mechanistic model of the relevant cytochrome P450 monooxygenase was proposed: an irregular polyhedron containing two hydrophilic patches [8, 9]. Recently, 11α-hydroxylase of *R. oryzae* was expressed in recombinant yeast and used in its pure form to transform progesterone, testosterone, 11-deoxycorticosterone, and 11-deoxycortisol into varying amounts of 11α-hydroxyprogesterone and 6β-hydroxyprogesterone [10]. This result provides strong support for the “one enzyme, multiple substrate orientations” hypothesis. Stereochemical restrictions were also invoked to rationalize the diverse metabolites of steroids in cultures of *Aspergillus tamarii* [11–13]. Our studies indicated that this mechanism may also occur in steroid hydroxylation reactions carried out by cultures of the filamentous fungi *Beauveria bassiana*, *Absidia coerulea*, and *Mortierella isabellina* [14–16].

Our knowledge of the diversity of metabolic routes of steroids is not, however, complemented by a deep understanding of enzymatic mechanisms of action on steroid substrates. In particular, structural information on the relevant enzymes is scarce. There are only a few reports in the Protein Data Bank (PDB) on crystal structures of bacterial Baeyer–Villiger monooxygenases (BVMOs) or dehydrogenases [17–21], and only a fraction of those relate to enzymatic action on steroids [17, 18]. The first structural report on fungal BVMO appeared in mid-2016 [22], and describes an enzyme from *Aspergillus flavus* that converts alkanones and aryl ketones to esters; its activity towards cyclic ketones is much lower. On the other hand, mammalian steroid receptors have received some attention from researchers, which has resulted in the characterization of the hydrogen-bonding network that allows the binding of steroids by the human mineralocorticoid receptor (MR) protein NR3C2 [23], for example.

**Fig. 1:**
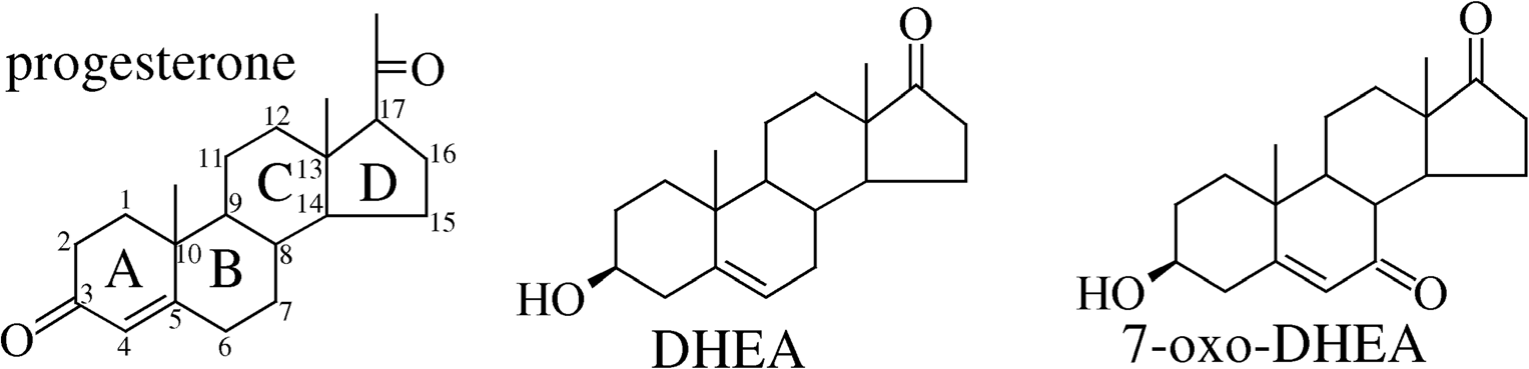
Structures of the steroids used as ligands in this work. The atom numbering scheme and labels *A–D* for the rings of the steroid nucleus are shown for progesterone

Our interest in the substrate specificity of enzymes acting on steroids prompted us to carry out the study reported in the present paper, which tackled the subject using docking and all-atom molecular dynamics (MD). We chose to investigate the orientational versatility of steroidal ligands in the binding pockets of two proteins: the human mineralocorticoid receptor MR [23] and the bacterial BVMO from *Rhodococcus rhodochrous* [17]. The selected ligands were progesterone (an important hormone that was experimentally found to bind strongly to the MR and was transformed by the chosen BVMO) as well as DHEA together with its 7-oxo derivative (see Fig. 1); the latter two ligands are of special interest to us (we have already studied them in previous investigations [3, 24]). In the following, we report on the docked structures of these three steroid ligands, their relative energies (scoring functions), as well as the dynamic behavior of the steroids in the binding pockets of the receptors.

## Methods

The initial structures of the proteins were taken from the PDB repository [25]; the structural entries 2AA5 (for the MR protein [23]; this contains progesterone as the ligand) and 4AOS (for the steroidal BVMO [17]; loaded with cofactors only, not the steroid substrate) were used. The residue numbers stated subsequently in this paper correspond to the numbering schemes used for the PDB entries. The files were manually edited to remove duplicate atoms resulting from crystal disorder, and only one chain of the dimeric 2AA5 structure was retained. The structures of the three ligands (see Fig. 1) were based on the progesterone skeleton of the MR–steroid complex 2AA5. After performing chemical modifications, the relevant hydrogen atoms were added and the structures were optimized at the semiempirical AM1 level. Further, the topology and parameter files were generated for the ligands with the GAFF force field [26], and the atomic charges were parameterized with the AM1-BCC scheme. This part of the study as well as the subsequent molecular dynamics simulations were carried out with the AMBER14 + AmberTools 2015 suite [27].

Docking simulations were performed with the Autodock Vina 1.1.2 package [28]. For the MR protein, the center of the cubic docking box was placed at the barycenter of the ligand, and the box edge was set to 24 Å, meaning that the box covered ca. 75% of the volume of the single protein subunit. For the BVMO (for which the ligand was not included in the crystal), the center was placed at the barycenter of the protein, close to the estimated binding site and the NADP and FAD cofactors, and the box edge was set to 48 Å, meaning that the box included all of the protein. The docking runs were repeated five times for each of the six protein–ligand pairs.

For the MR protein (for which the exact ligand position was known), a full-atom molecular dynamics simulation was carried out. The aim was to reproduce the dynamics of the ligand within the protein, so the simulation length scale was adapted to the dynamics of the hydrogen-bond network rather than to the protein relaxation times. The initial structures were the structures with docked ligands.

In the first step, using the utilities package in AmberTools 2015, hydrogen atoms and missing heavy atoms were added to the structure of the monomeric protein. The protein–ligand complex was then immersed in a rectangular box of TIP3P water with a 12-Å buffer, and three Na^+^ ions were added at the locations of minimum electrostatic potential to ensure neutrality. The force fields used for the ligand and the protein were GAFF [26] and ff14SB [29], respectively. The simulation then progressed as follows: 1000 steps of steepest-descent energy minimization were performed to remove bad contacts before 30 ps of NVT thermalization and 70 ps of NPT equilibration (*T* = 300 K, *p* = 1 atm) were carried out. The production run was 1 ns of NVT simulation. A 1-fs timestep was used consistently to propagate the nuclear degrees of freedom. The particle mesh Ewald technique was used for electrostatic summations, and the direct space nonbonded cutoff was set to 12 Å. Bonds involving hydrogen atoms were restrained with the SHAKE algorithm [30]. The MD runs were carried out in triplicate for each binding mode found for a given ligand (two for progesterone and three each for DHEA and 7-oxo-DHEA), starting with different random number seeds. No statistically significant differences were observed among the triplicates performed for a given run.

These calculations performed with the AMBER14 suite were followed by trajectory analysis. The VMD 1.9.1 program [31] was employed for this purpose, as well as for the structure visualization.

## Results and discussion

### Docking simulations

**Fig. 2:**
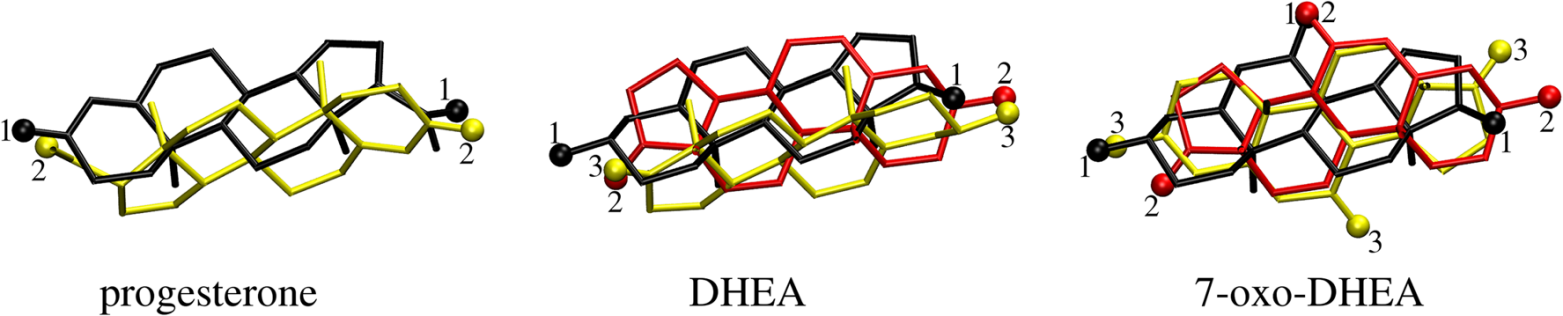
Docking results for the MR protein and three steroid ligands. Oxygen atoms are represented as *spheres*; hydrogen atoms are omitted for clarity. The *labels on the oxygen atoms* indicate the rank of the structure, 1 being the highest ranked (i.e., it shows the strongest affinity); see Table 1

We first attempted to dock the three steroids to the human mineralocorticoid receptor NR3C2, because the exact position of the steroid ligand at this receptor has already been determined experimentally [23]. Progesterone binds strongly to this protein without triggering its regulatory activity [23]. The repeated docking runs always yielded the same two structures of MR-docked progesterone, as depicted in Fig. 2. The best of those structures (i.e., the structure with the highest affinity score, −12.1 kcal/mol) corresponds to the experimental position of the ligand; the root mean square deviation (RMSD) between the experimental and best MR-docked position of progesterone is 0.23 Å. For DHEA and 7-oxo-DHEA, the best docked structures are again very similar to the experimental orientation of progesterone; the corresponding RMSD (calculated for the steroid nucleus atoms only) is 0.39 Å for both DHEA and 7-oxo-DHEA. Some of this increase in RMSD for DHEA and 7-oxo-DHEA compared to progesterone can be attributed to differences in the steroid nuclei of these ligands (different locations of the double bond). However, the calculated affinities are smaller for DHEA and 7-oxo-DHEA: −10.8 kcal/mol and −10.9 kcal/mol, respectively. The negligible difference in affinity between DHEA and its derivative could be due to the weak interaction (3.4 Å) between the carbonyl oxygen at C-7 of the ligand and the sulfur atom of Met852 (as mentioned above, the residue labels follow the original numbering schemes for the PDB entries). The loss of affinity in comparison to progesterone is caused by the fact that the side-chain oxygen atom of the latter forms a hydrogen bond with Thr945–OH (2.72 Å), while the acetyl sidechain is replaced with a keto function in DHEA and its derivative, and the corresponding O···O distance increases to 3.8 Å. That said, the primary binding mode is conserved for the three investigated steroids.

It is important to note that other binding modes were located by the docking procedure (see Fig. 2), and their calculated affinities and orientations (see Table 1) follow an interesting pattern. The orientations are labeled according to the ideas of Brannon et al. [7] and Holland et al. [6]. We denote the highest-affinity structure as the “normal” (n) structure, which corresponds to the experimentally observed structure docked to the MR protein. When the steroid nucleus is docked such that the A and D rings have switched positions with regard to the normal orientation, it termed “reversed” (r) binding. If the methyl groups at C-10 and C-13 point in the opposite directions to their directions in the normal case, we denote this “inverted” (i) binding. Finally, if both reversed and inverted binding occur at the same time, the result is “reverse inverted” (ri) binding. In some cases, the angle between the normal direction of the methyl groups and the considered structure is closer to 90° than 180° and the rings are reversed, leading to (r/ri) binding.

**Table 1:** Binding affinities (in kcal/mol) and orientations of the binding modes of progesterone, DHEA, and 7-oxo-DHEA to the two studied proteins (MR NR3C2 and steroid BVMO)

Protein	MR NR3C2			Steroid BVMO		
Ligand	Progesterone	DHEA	7-Oxo-DHEA	Progesterone	DHEA	7-Oxo-DHEA
Rank 1	−12.1 (n)	−10.8 (n)	−10.9 (n)	−8.6 (n)	−7.9 (n)	−7.8 (r)
Rank 2	−8.9 (r/ri)	−9.1 (ri)	−9.2 (ri)	−7.3 (r)	−7.8 (r)	−7.6 (n)
Rank 3	–	−9.0 (r/ri)	−8.4 (i)	−6.9 (i)	−7.4 (i)	−7.3 (ri)
Rank 4	–	–	–	–	−7.1 (ri)	−7.3 (i)

The ranks of the docked structures correspond to the labeling in Fig. 2 for MR NR3C2. Orientation codes: *(n)* normal, *(r)* reverse, *(i)* inverted, *(ri)* reverse inverted (see text for details). Results are from Vina docking runs

Continuing the discussion of the results obtained for the MR protein, we note that only two orientations were found for the progesterone ligand. The normal (experimental) one is stabilized by two hydrogen bonds between the A-ring hydroxyl and the Arg81 amine nitrogen (2.80 Å) and between the D-ring side-chain oxygen and the Thr203 hydroxyl (2.74 Å). In the (r/ri) orientation, these contacts (now between the D-ring side chain and Arg81 and between the A-ring OH and Thr203) are, respectively, 3.92 Å and 2.80 Å long. The loss of the first contact significantly worsens the affinity, so we would expect the progesterone to bind to the MR protein in a specific orientation. On the other hand, DHEA, which has no acetyl sidechain but does have an oxo function at C-17, potentially binds as strongly in all three modes. The preferred (n) mode has close contacts with Arg81 (2.88 Å) and Gln40 (2.84 Å), but the contact to Thr203 is lost (>4 Å). The (ri) mode regains this contact (2.70 Å), as also does the (r/ri) mode (2.60 Å), but these modes lose contact with Arg81. They do, however, retain the hydrogen bonds to Gln40 (2.67 Å for (ri) and 3.37 Å for (r/ri), making it the least preferred orientation). Thus, DHEA probably binds in more than one preferred orientation. Similar behavior is seen for 7-oxo-DHEA, but the two best orientations, (n) and (ri), also form a short contact (3.3 Å) with the sulfur atom of Met116. The mode with the lowest affinity also lacks the contact with Thr203. The dynamic variability of these contacts was subsequently investigated further by all-atom MD. Here we conclude by stating that even relatively small structural modifications (i.e., DHEA vs. 7-oxo-DHEA) can influence the network of contacts formed and change binding preferences.

**Fig. 3:**
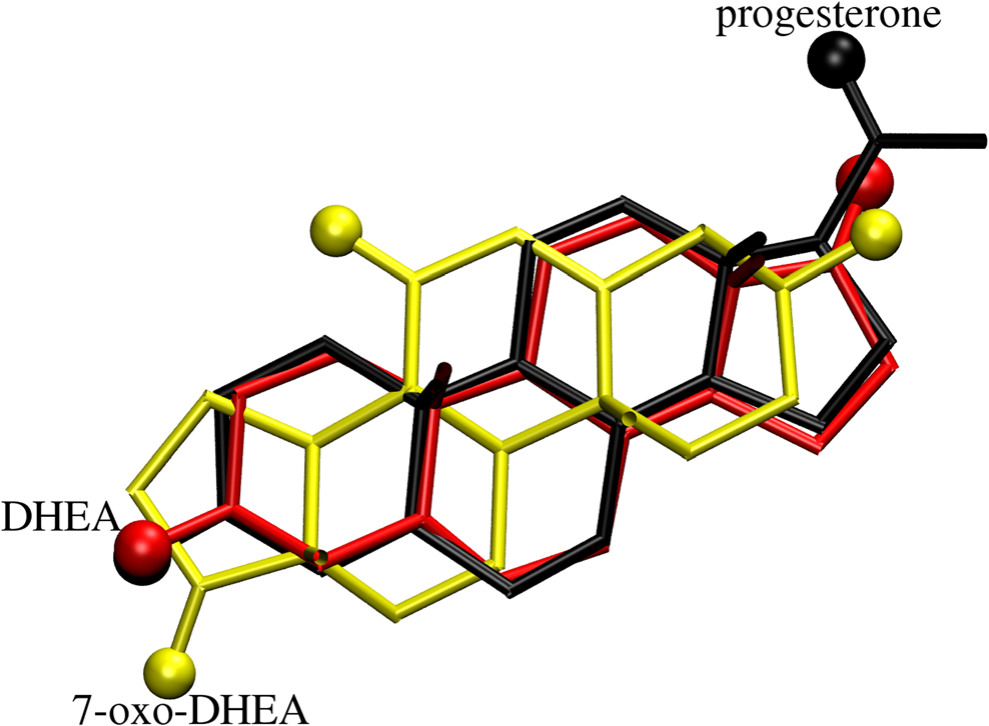
Best binding modes of the three steroid ligands within the steroidal BVMO. Oxygen atoms are represented as *spheres*; hydrogen atoms are omitted for clarity

In the case of the steroidal BVMO from *Rhodococcus rhodochrous* [17], there is no reference experimentally derived position of the steroid. However, the progesterone and DHEA bind at the same location and with the same mode, whereas 7-oxo-DHEA chooses the same location but the (r) orientation with respect to the other ligands (see Fig. 3). Thus, we chose to label the orientations with respect to the best (n) docking results for progesterone and DHEA. Note that the (n) binding mode of 7-oxo-DHEA is only 0.2 kcal/mol worse than its best (r) mode, so the ranking in this case is rather unclear. Indeed, this is a general trend that started to emerge for the MR protein but is readily apparent in the BVMO case: while progesterone has a preferential binding mode (n) and its other orientations are clearly less preferred, the affinities of the binding modes occur within a much narrower range for both DHEA and its derivative: the best and worst binding modes are separated by only 0.8 kcal/mol for DHEA and just 0.5 kcal/mol for 7-oxo-DHEA. This is consistent with previous reports on the diversity of the products of enzymatic transformations of steroids [10–16].

The steroids bind to the BVMO loaded with NADP and FAD cofactors through the following contacts observed for the best binding modes. Progesterone in its (n) orientation forms hydrogen bonds to Ser54–OH (3.10 Å) and Tyr61–OH (3.08 Å) via its acetyl oxygen function. Additional stabilization is gained through a hydrophobic interaction with Trp62. Longer, weaker contacts (>4 Å) are formed with Arg123, Asn160, and His184, as well as with the FAD cofactor. The DHEA molecule in its (n) orientation retains the hydrophobic interaction and the contacts with Tyr61 (3.85 Å), Arg123, Asn160, and His184; a contact with Lys404–NH_3_^+^ (3.86 Å) is also formed. The 7-oxo-DHEA molecule is also oriented parallel to the Trp62 residue, and it forms hydrogen bonds to Asn160 (3.19 and 3.36 Å), the Ala53 backbone N (3.27 Å), and the FAD cofactor (3.04 Å). The latter two bonds involve the 7-keto oxygen atom and may be responsible for the small preference for the (r) orientation over the (n) one.

Summarizing the results of the docking study, it is clear that DHEA and 7-oxo-DHEA are both more flexible ligands than progesterone (this behavior was observed with both of the investigated proteins). Especially for the steroid BVMO, a mixture of oxidation products should be expected. However, docking presents a simplified and static picture. Therefore, we followed the docking study with an investigation of the dynamics of the steroid–host contacts.

### Molecular dynamics simulations

**Fig. 4:**
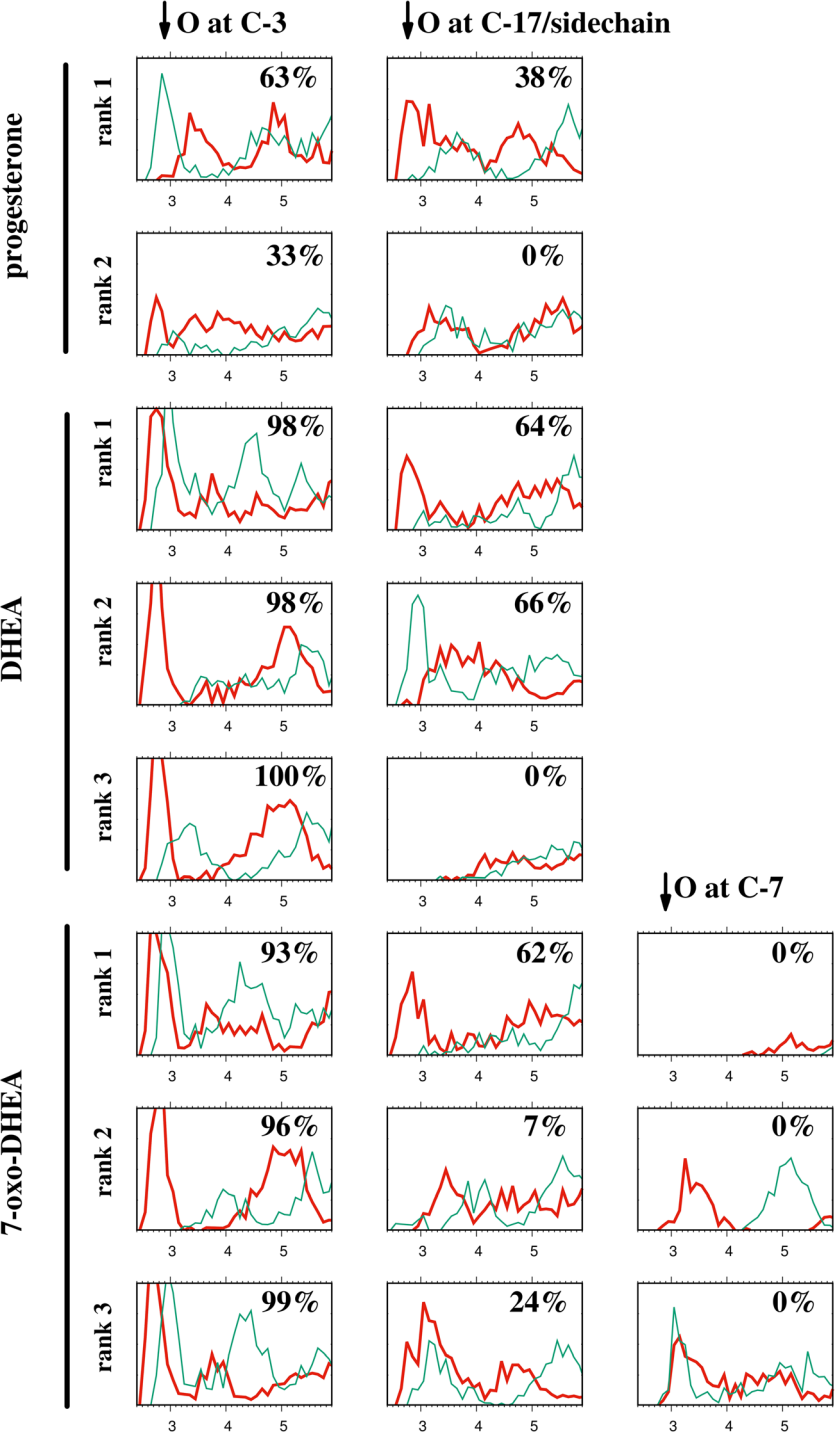
Radial distribution functions (RDFs) for the indicated oxygen atoms of the steroid ligands (results from MD simulation). Chart axes: *x*-axis is the O···O/O···N distance in Å; *y*-axis is the normalized RDF in units of Å^−1^. *Thick lines* show O···O RDFs and *thin lines* show O····N RDFs. The *percentages* refer to populations of the hydrogen bonds (O···O and O···N combined) between the steroid oxygen function and the protein

The mineralocorticoid receptor NR3C2 was found in the docking study to bind progesterone preferentially in one orientation. The affinity of the preferred orientation is 3.2 kcal/mol higher than that of the other binding mode identified in the docking study. However, the differences in binding-mode affinity are smaller for DHEA and 7-oxo-DHEA, and these ligands can adopt three orientations, although the best orientation is consistent with that for progesterone. These facts are reflected in the dynamics of the hydrogen-bonding network around the ligand. The steroids considered here possess up to three oxygen functions (see Fig. 1), namely those at C-3, at C-17 (or the C-17 side chain), and at C-7 (7-oxo-DHEA only). Due to the large number of possible contacts, we initially chose to represent the results of the MD simulations in the form of radial distribution functions (RDFs), and we later complemented those RDFs with a hydrogen-bonding analysis. For each of the ligand oxygen atoms, two such RDFs were calculated, relating to contacts with protein oxygen and nitrogen atoms. The results are shown in Fig. 4.

Comparison of the results for the best (rank 1) “normal” orientations of progesterone, DHEA, and 7-oxo-DHEA shows differences between their hydrogen-bonding networks, which are due to differences in the nature of the oxygen function at C-3: this is a carbonyl oxygen for progesterone and a hydroxyl function for DHEA and its derivative. The C-3 carbonyl oxygen atom in progesterone prefers to form contacts with protein nitrogen atoms (RDF maximum at 2.85 Å), while there are contacts with both oxygen (2.70–2.75 Å) and nitrogen (2.95 Å) atoms for the other two steroids. While it is true that O···N hydrogen bonds are generally weaker than their O···O analogs [32], in this case there are charge-assisted hydrogen bonds with charged amino functions of arginine. Continuing the discussion of the (n) orientation (rank 1), we note that—contrary to the docking result—the difference between the C-17 substituents (acetyl in progesterone and carbonyl oxygen in DHEA and 7-oxo-DHEA) does not lead to differences in the distance of the contact with the oxygen at C-17/side chain. The corresponding RDFs are similar and show maxima at 2.7–2.9 Å. This is another manifestation of the adaptability of various steroids to binding pockets.

When less stable binding modes are considered, there are two possible cases. First, the rotated steroid molecule can lose its most important contacts. This is the case for the (r/ri) orientation of progesterone (rank 2), which loses interactions with nitrogen atoms. Even though the carbonyl oxygen at C-3 assumes the role of the acetyl oxygen in the (n) orientation (sharp maximum in the O···O RDF close to 2.7 Å), the acetyl oxygen of the rank-2 structure has its RDF maxima at distances larger than 3 Å. Thus, the (r/ri) orientation for progesterone is significantly destabilized. The same holds for the rank-3 structure (r/ri) of DHEA, for which the oxo function at C-17 has no hydrogen-bonded contacts. A second type of behavior occurs for the rank-2 structure of DHEA (ri) and the rank-3 structure of 7-oxo-DHEA (i). In these cases, the oxygen atoms are able to assume the roles of their counterparts in the “normal” rank-1 structure. Compare, for example, the RDFs for the rank-1 and rank-2 structures of DHEA. Contact with nitrogen atoms at 2.95 Å is present for the C-3 hydroxyl and carbonyl oxygen atoms in the rank-1 and rank-2 structures, respectively. Contact with oxygen atoms at 2.70–2.75 Å is present for the C-17 carbonyl and C-3 hydroxyl oxygen atoms in the rank-1 and rank-2 structures, respectively. The rank-2 (ri) structure of 7-oxo-DHEA lies between the two behavioral types described above: while its C-17 carbonyl oxygen does not form short contacts (<3 Å), its C-7 oxo function interacts much more strongly with protein oxygen atoms than does the C-7 oxygen in the rank-1 structure.

RDF analysis is not able to differentiate clearly between hydrogen bonds and short electrostatic contacts. Hydrogen bonds are, however, regarded as the tools that enzymes use to activate substrates [33]. It is generally accepted that low-barrier hydrogen bonds (LBHBs) are formed as an enzyme’s substrate approaches the transition structure [33]. However, the NR3C2 protein is a steroid receptor, not an enzyme, so it does not require strong, short hydrogen bonds to form between the substrate and the host. Indeed, the distances of the contacts located in the RDFs are mostly within 2.7–3.0 Å. However, as this study was intended to initiate further computational research by us in the field of steroid enzymatic catalysis, we chose to proceed with a more detailed analysis of the hydrogen bonds along the MD trajectory. Numerous indicators of the hydrogen bonding have been proposed, but geometric criteria are the most practical for structural protein research. Commonly, a donor–acceptor cutoff of ca. 3.0 Å, based on the sum of the van der Waals radii [34], is used to detect O···O hydrogen bonds in small-molecule X-ray diffractometry. Much more relaxed criteria have been proposed for experimental studies on proteins, including *r*(D···A) < 3.9 Å and ∠(D–H···A) > 90° [35]. This relaxation of the criteria arises, at least partially, from the fact that an enzyme can undergo large structural changes when it is in action (see [36] for a recent example), and the network of hydrogen bonds also strongly fluctuates. Considering the dynamic nature of the contacts formed, we finally chose to use rather conservative values to define the occurrence of hydrogen bonding: *r*(D···A) < 3.3 Å and ∠(D–H···A) > 135°. Proper care was taken to treat steroid oxygen functions as either acceptors only (keto function) or possible donors and acceptors (hydroxyl function). The hydrogen-bond populations (the percentage of the MD trajectory that satisfies the abovementioned criteria) for the oxygen functions of the steroids are indicated in Fig. 4. It is noticeable that the best (rank-1) binding modes are associated with the largest populations for all three of the investigated ligands. Comparison with the RDF results shows that there are cases with short contacts that are not regarded as hydrogen bonds, especially for the keto function at C-7 in 7-oxo-DHEA. These correspond to contacts between two sites that cannot act as donors (e.g., the keto function of the steroid and the carbonyl function of the protein backbone). The flexibility of steroids as ligands is clearly demonstrated by DHEA, as the rank-1 and rank-2 binding modes are characterized by virtually identical hydrogen-bond populations. Comparison of the RDFs shows that the secondary interaction switches from the O···O (64% population) to the O···N (66% pop.) type.

The above discussion of the MD results focused on hydrogen bonding and electrostatic contacts. This was done to rationalize the possibility that the hydrogen-bonding network is preserved to some extent in the inverted and reversed binding modes. Also, the presence of sharp maxima indicates that the steroid ligands do not reorient in the binding pocket on the investigated timescale. Note that, since we did not calculate the MD-based free energies of binding, we were not able to compare the docking results with the MD results directly. An interesting case could be the rank-3 structure of 7-oxo-DHEA. Although it is the worst structure identified by docking, it seems to have a comparable hydrogen-bonding network to the rank-1 binding mode. Therefore, we are currently carrying out a detailed MD study of the behavior of DHEA and its derivatives in the MR protein and in the BVMO enzyme.

## Conclusions

Recent advances in structural studies of steroid-binding proteins—including a notable increase in the number of known 3D structures of steroid–protein complexes—have resulted in the possibility of investigating the behavior of steroid ligands through mechanistic simulation. The results of the combined docking and molecular dynamics study of three steroids bound to two different proteins (a mineralocorticoid receptor and a bacterial monooxidase) performed in this work fully support the notion that bound steroidal ligands possess considerable orientational versatility. At least two binding modes were found for each steroid studied in this work, and their affinities do not differ dramatically. Moreover, the MD study of the MR protein indicated that changes in orientation do not automatically result in serious disruption of the hydrogen-bonding network stabilizing the ligand. Especially for DHEA and 7-oxo-DHEA, the oxygen functions were found to be adaptable in terms of their intermolecular contacts. This adaptability was less visible for progesterone. Our study thus provides computational support for a possible explanation for the diversity of products of enzymatic transformations of steroid substrates.
